# Optimization and validation of ELISAs for interferon-gamma determination in bison

**DOI:** 10.1177/10406387251344567

**Published:** 2025-06-03

**Authors:** Josephine Chileshe, Todd Shury, Jeffrey M. Chen

**Affiliations:** Vaccine and Infectious Disease Organization (VIDO), University of Saskatchewan, Saskatoon, Saskatchewan, Canada; Parks Canada Agency, Government of Canada, Saskatoon, Saskatchewan, Canada; Vaccine and Infectious Disease Organization (VIDO), University of Saskatchewan, Saskatoon, Saskatchewan, Canada

**Keywords:** assay validation, bison, ELISA, interferon-gamma, *Mycobacterium bovis*

## Abstract

Bovine tuberculosis, caused by *Mycobacterium bovis*, is endemic in the Wood Buffalo National Park, Canada, home to free-ranging and threatened wood bison. This disease poses a threat to the conservation of this culturally and ecologically important animal species, as well as potentially impacting the health of humans and other animal species via zoonosis and spillover, respectively. The ability to detect infection early will minimize and prevent the potential risk of *M. bovis* transmission. Interferon-gamma (IFNγ) assays are a reliable detection method for *M. bovis* in cattle and other wildlife species and may have diagnostic value in bison as well. We aimed to optimize and partially validate 2 commercial IFNγ ELISAs to detect endogenous bison IFNγ in mitogen-stimulated whole blood. Parameters evaluated included antibody identification, sample matrix effect, dilution linearity, assay reproducibility, and limit of quantification. The optimized assays demonstrated linear responses to recombinant bovine and endogenous bison IFNγ (range: 1–125 pg/mL; *R*^2^ = 0.99), with good recovery and fair reproducibility, and a low limit of quantification of 1 pg/mL. Mabtech bovine Flex and Pro kits have the same antibodies but in 2 different assay formats; an in-house assay platform (Flex kit) and precoated plates (Pro kit) are considered suitable for measuring bison IFNγ, offering flexibility depending on available resources.

In Wood Buffalo National Park (WBNP; Alberta, Northwest Territories, Canada), bovine tuberculosis (bTB), caused by *Mycobacterium bovis*, is endemic in free-ranging but threatened wood bison (*Bison bison athabascae*),^9^ and these bison populations are considered to be maintenance hosts of *M. bovis*.^15^ This infectious disease poses a serious threat to the health and conservation of this important species, as well as impacting the health of human and livestock populations, especially in communities around WBNP.^7^

The immune response to mycobacterial infection is predominantly cell-mediated immunity (CMI), and antemortem tests are globally used for the detection of this response.^20^ In Canada, the “standard diagnostic” test for bTB is the tuberculin skin test, which evaluates the cell-mediated immune responses to tuberculin purified protein derivative.^4^ However, the application of the tuberculin skin test in bison under field conditions poses significant challenges. These include low sensitivity (50–92%) and specificity (34–100%),^13^ as well as logistical difficulties associated with the need to re-immobilize bison to measure changes in skin thickness due to inflammation.^4^ Therefore, research focusing on identifying alternative immunoassays for CMI response in wood bison that can be used in the field to support bTB management in Canada is necessary. An efficient CMI response is dependent on T cells, and interferon-gamma (IFNγ), the main cytokine produced by T cells in this response, is an important biomarker used in the diagnosis of mycobacterial infections in humans,^16^ cattle,^10^ and wildlife.^5,12,18^

However, available immunoassays that detect and measure IFNγ are limited for use in wildlife species due to the requirement for specific reagents or the lack of validated tests.^23^ Notably, bovine-specific IFNγ antibodies are produced commercially and have been reported to cross-react with other species in the *Bovidae* family, such as African buffalo^18^ and European buffalo.^8^ These findings support the use of commercial reagents to develop bison immunoassays. In addition, these immunoassays are especially important for wildlife species that are indigenous, protected, and valuable, and when postmortem samples are unavailable. We aimed to test and partially validate commercial bovine IFNγ ELISA kits to measure bison IFNγ in mitogen-stimulated blood. The partially validated assay could further be evaluated as a potentially more sensitive antemortem detection test for *M. bovis* infection in bison herds.

## Materials and methods

### Animals

Whole blood samples were collected from 4 healthy, bTB-free cattle and 4 bison in 6-mL lithium–heparin vacutainers (BD Biosciences) during routine handling procedures. These samples were obtained as part of other approved research projects granted by the Animal Research Ethics Board of the University of Saskatchewan (Animal Use Protocols 20220089 for cattle and 20190085 for bison). Cattle blood samples were sourced from the VIDO research farm in Saskatoon; bison samples were collected from the Native Hoofstock Centre at the University of Saskatchewan, Saskatoon.

### Whole blood stimulation

For each animal, 1.5-mL aliquots were transferred to wells of a non-treated sterile flat-bottom 24-well tissue culture plate (Corning). Pokeweed mitogen (PWM; Prionics, ThermoFisher) diluted in RPMI medium (MilliporeSigma) was added to wells at a final concentration of 5 µg/mL; RPMI medium alone was added to the other wells as a negative control. The wells were designated as PWM and nil, respectively, and then incubated for 24 h at 37°C. Plasma samples from individual animals were harvested and, additionally, pooled sample aliquots were created with sufficient volume for repeated experiments and sampling and then aliquoted in 1.5-mL tubes to avoid freeze–thaw between experiments and stored at −80°C until analyzed.

### Screening of commercial anti-IFNγ antibodies

Commercial IFNγ ELISA platforms were tested for measuring IFNγ in bison and cattle plasma. These antibodies were selected for the broad range of species that they cross-react with (specificity) as indicated in the manufacturer’s datasheet: 1) Flex bovine ELISA kit [ELISA Flex: bovine IFNγ (HRP), 3119-1h-6, batch 29; Mabtech] contains a matched pair of monoclonal antibodies (capture antibody: MT17.1; detection antibody: MT307-biotin) specific for bovine IFNγ and cross-reacts with IFNγ from sheep; 2) Flex llama ELISA kit [ELISA Flex: llama IFNγ (HRP), 3123-1h-6, batch 2; Mabtech] contains a matched pair of monoclonal antibodies (capture antibody: bIFNγ-I; detection antibody: PAN, biotin) specific for bovine IFNγ and cross-reacts with IFNγ from llama, alpaca, sheep, horse, and dog; and 3) bovine IFNγ specific ELISA kit (MCA5638KZZ; Bio-Rad) contains a matched pair of monoclonal antibodies (capture antibody: MCA5638KZZA; detection antibody: MCA5638KZZC) specific for bovine IFNγ (UniProt: P07353) and cross-reacts with IFNγ from African buffalo,^3^ sheep, and goat.^21^

#### Mabtech kits

The ELISA protocols were performed according to the manufacturer’s instructions. Capture antibodies (Mabtech) were diluted to 2 µg/mL in 1× PBS (Gibco; ThermoFisher). The 96-well microtiter plates (Corning) were coated by adding 100 μL/well of diluted capture antibody of each pair and incubating the plates overnight at 4°C. The following day, the solution containing the capture antibody was removed, and wells were blocked with 200 μL of blocking buffer, also referred to as incubation buffer [PBS with 0.05% Tween 20 and 0.1% bovine serum albumin (BSA); lot QA011010, Bio Basic]. The plates were incubated at room temperature (RT) for 1 h and then washed 5 times (300 µL/well) with wash buffer solution (1× PBS with 0.05% Tween 20; MilliporeSigma). After washing, pooled PWM plasma samples were diluted 1:2 in ELISA diluent (code 3652-D2; Mabtech), and 100 µL was added to each well in duplicate. The plates were incubated at RT for 2 h and then washed 5 times. The bovine detection antibody was diluted to 0.25 µg/mL in incubation buffer, and to 0.2 µg/mL for the llama kit; 100 µL was added to each well, and both plates were incubated at RT for 1 h. After incubation, the plate was washed 5 times, and 100 μL/well of streptavidin–horseradish peroxidase (HRP; Mabtech) diluted 1:1,000 in incubation buffer was added to both plates and incubated at RT for 1 h. After washing the plates 5 times, 100 μL of colorimetric tetramethylbenzidine (TMB, code 3652-F10; Mabtech) enzyme substrate was added to each well and incubated at RT for 15 min (protected from direct light). The reaction was stopped by adding 100 μL of 2M H_2_SO_4_ solution to each well. The optical density (OD) of each test and control well was measured at the specific wavelength of 450 nm and reference wavelengths of 570–650 nm, of which 630 nm was selected as the reference wavelength (SpectraMax i3x ELISA microplate reader with SoftMax Pro software; Molecular Devices). Results were calculated as the OD value measured at 630 nm subtracted from that measured at 450 nm; negative assay controls were used to ensure that the test well signal was specific to the PWM plasma sample and normalized OD values.

#### Bio-Rad kit

The ELISA protocol was performed according to the manufacturer’s instructions. A 1:200 dilution of the bovine IFNγ capture antibody in coating buffer (supplied) was prepared, and a 96-well microtiter plate (supplied) was coated by adding 100 μL/well of diluted coating antibody and incubating the plates overnight at 4°C. The following day, the plates were washed 3 times (300 µL/well) with wash buffer (0.2 M NaCl, 0.05% Tween 80; MilliporeSigma). Blocking buffer (200 μL of 1× PBS with 4% bovine serum albumin, lot QA011010; Bio Basic) was added to block each well and incubated at RT for 1 h. After washing, the pooled PWM plasma samples were diluted 1:2 in wash buffer, and 100 µL was added to each well in duplicate and incubated at RT for 1 h, then washed 3 times. The detection antibody was prepared at 1:500 dilution in the wash buffer and incubated at RT for 1 h. Thereafter, the plate was washed 3 times, and 100 μL/well of HRP (supplied) diluted 1:1,000 in wash buffer was added to the wells and incubated at RT for 1 h. After washing the plates 3 times, 100 μL of TMB (supplied) enzyme substrate was added to each well and incubated at RT for 10 min (protected from direct light). The reaction was stopped by adding 100 μL of 2M H_2_SO_4_ solution to each well. The ELISA results were calculated as the OD at 450 nm; negative assay controls were used to ensure that the test well signal was specific to the PWM plasma sample and normalized OD values.

### Selection of an ELISA kit

The pooled PWM cattle and bison plasma samples were diluted 1:2 and assayed in duplicate using the bovine (Flex kit) and llama IFNγ antibody pairs (products 3119 and 3123, respectively; Mabtech) as described above. In addition, these samples were assayed using 2 precoated kits: ELISA Pro (bovine IFNγ, 3119-1HP-1, batch 3; Mabtech) and Bovigam 2G TB kit (cat. 63330; ThermoFisher) that included supplied reagents used according to the manufacturer’s instructions. The ELISA platform with the highest detectable PWM signal (based on the mean OD of the test wells) was selected for further validation using bison plasma samples.

### Validation of IFNγ ELISA

The bovine Flex and Pro kits utilize the same bovine IFNγ antibodies but are designed for different platforms: the Flex kit is an in-house (Do-It-Yourself) platform, whereas the Pro kit is a pre-coated, ready-to-use platform. Their performance was characterized according to the manufacturer’s instructions. A 10-µL aliquot of recombinant bovine IFNγ (rIFNγ) standard solution (10 ng/mL) was diluted in 490 µL of incubation buffer to create a working solution of 500 pg/mL. This was serially diluted 1:2 (2-fold dilutions to produce a dilution series of 500–1 pg/mL. Results for both the Flex and Pro kits were measured and calculated as above; the relationship between OD and IFNγ concentration was analyzed using linear regression analysis using the standard curve to determine bison IFNγ concentrations (GraphPad v.9; Dotmatics).

To determine the recovery of rIFNγ in bison plasma matrix to ensure the absence of analytical error and interference in immunoassays,^17^ dilution linearity was performed to demonstrate that bison plasma sample with a spiked concentration can be diluted to a concentration within a working range and still give a reliable result. A pooled nil bison sample was spiked with 10 ng/mL of bovine rIFNγ to obtain a plasma sample with ~800 pg/mL. This sample was serially diluted (2-fold dilutions) to form a 5-point dilution series. Duplicate samples were assayed, and IFNγ concentrations were calculated as described above. Thereafter, recovery (%) of bovine rIFNγ was calculated as described previously^1^: ([rIFNγ] in test sample ÷ [rIFNγ] in reference sample) × 100%.

The linearity and parallelism of the ELISAs were determined using IFNγ concentrations of 1–125 pg/mL. The pooled PWM bison plasma sample was diluted in ELISA diluent to obtain a plasma sample with ~125 pg/mL measured endogenous IFNγ. This sample was serially diluted 1:2 in ELISA diluent to form a 6-point dilution series, using 500 pg/mL of rIFNγ. Samples were assayed in duplicate, plasma IFNγ concentrations were calculated as described above, and both dilution series were analyzed by regression analysis. The regression value of rIFNγ was characterized as the correlation coefficient (*R*^2^). To determine parallelism, the regression slopes for the rIFNγ and endogenous IFNγ were compared using the *F*-test (GraphPad v.9).

The limit of detection (LOD) and limit of quantification (LOQ) help determine the assay’s ability to detect the analyte’s minimum concentration accurately. The LOD is the lowest analyte concentration to be detected reliably and distinguished from a blank value; the LOQ is the lowest concentration at which the analyte can be accurately detected with precision.^1,24^

The LOD and LOQ were determined using 34 replicates of blank samples (incubation buffer) for both the Pro kit and Flex kit, and these were analyzed on the same plate as a dilution series of rIFNγ consisting of 3.9, 2, and 1 pg/mL. For the 34 replicates, the mean OD value and SD were calculated. The LOD OD was calculated as the x̄ + 3 SD, and the LOQ OD was calculated as the x̄ + 10 SD as described previously.^2,6,17^

Reproducibility was evaluated using plasma from 4 bison. For each animal, PWM samples were diluted 1:32 in a pooled nil bison plasma sample and assayed in triplicate on the same microtiter ELISA plate, as above. This was repeated on 3 different days. Intra-assay precision was calculated as the CV of the results for the 3 replicates on each day. Inter-assay precision was calculated as the CV of the results of the 3 different days as described previously.^5^

Last, we compared the BSA concentrations (0.1% and 4%) in the blocking method, also referred to as incubation buffer, to ensure reproducible and reliable results. To achieve this, pooled PWM plasma samples from bison and cattle were assayed using the Bio-Rad bovine IFNγ ELISA kit as described above, following the manufacturer’s protocol with a 4% BSA concentration incubation buffer. These samples were also assayed using a modified protocol, in which the incubation buffer consisted of 1× PBS with 0.1% BSA. The ELISA results were calculated as the OD measured at 450 nm; negative assay controls were used to ensure that the test well signal was specific to the PWM plasma sample and normalized OD values.

## Results

**Figure 1.**
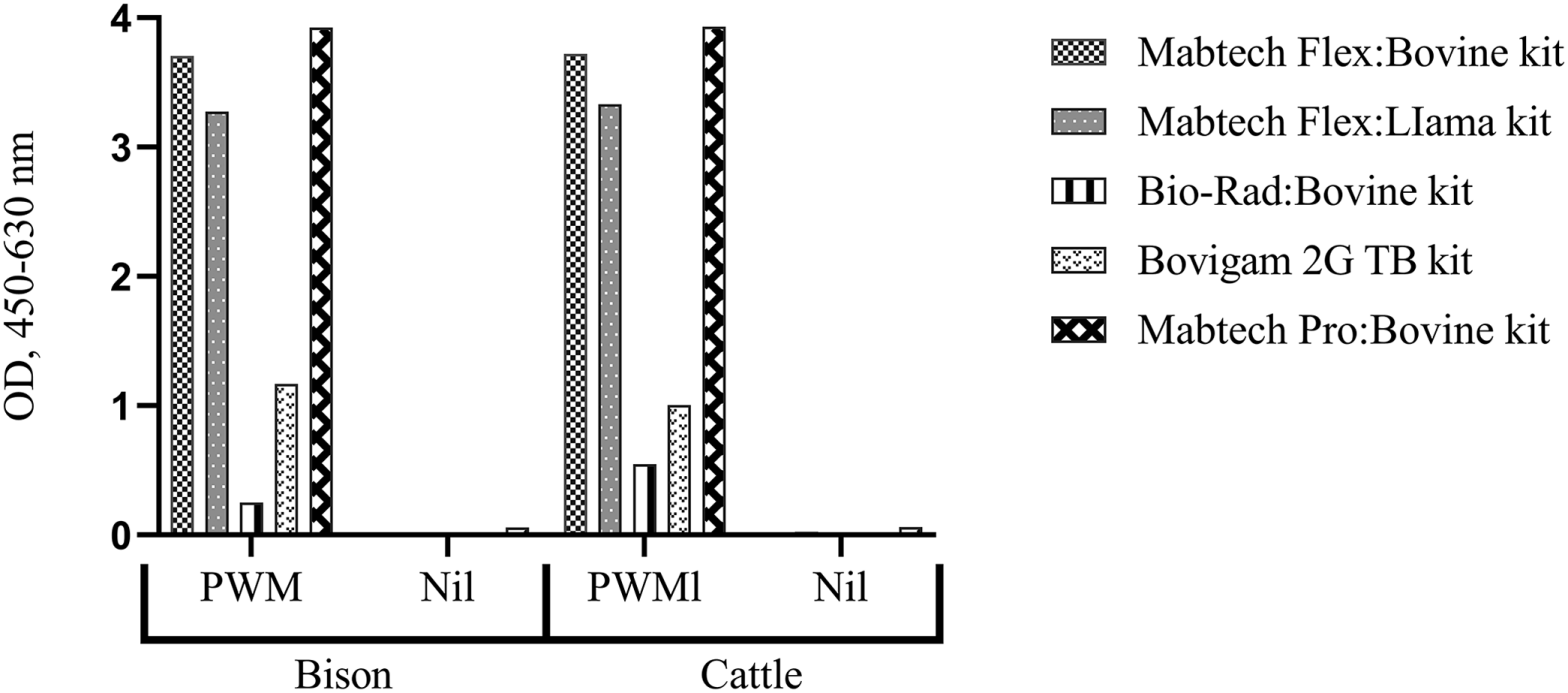
The comparative sensitivity of selected ELISAs for bison interferon-gamma (IFNγ). Whole blood from 4 bison and 4 cattle was incubated overnight with pokeweed mitogen (PWM; 5 µg/mL) and unstimulated (nil). Pooled PWM and nil plasma samples were then diluted 1:2 and assayed using the 5 commercial IFNγ ELISAs. The Flex and Pro kits had the highest optical density (OD).

Of the 3 antibody pairs screened, the Flex pair had the strongest signal (based on the mean OD of test wells) when assaying PWM-stimulated plasma of bison and cattle. In addition, compared with the 2 precoated ELISAs, the Pro had the strongest signal when assaying PWM-stimulated plasma samples of bison and cattle (Fig. 1). Therefore, the Flex and Pro kits were selected for validation analysis, but using bison plasma samples.

**Figure 2.**
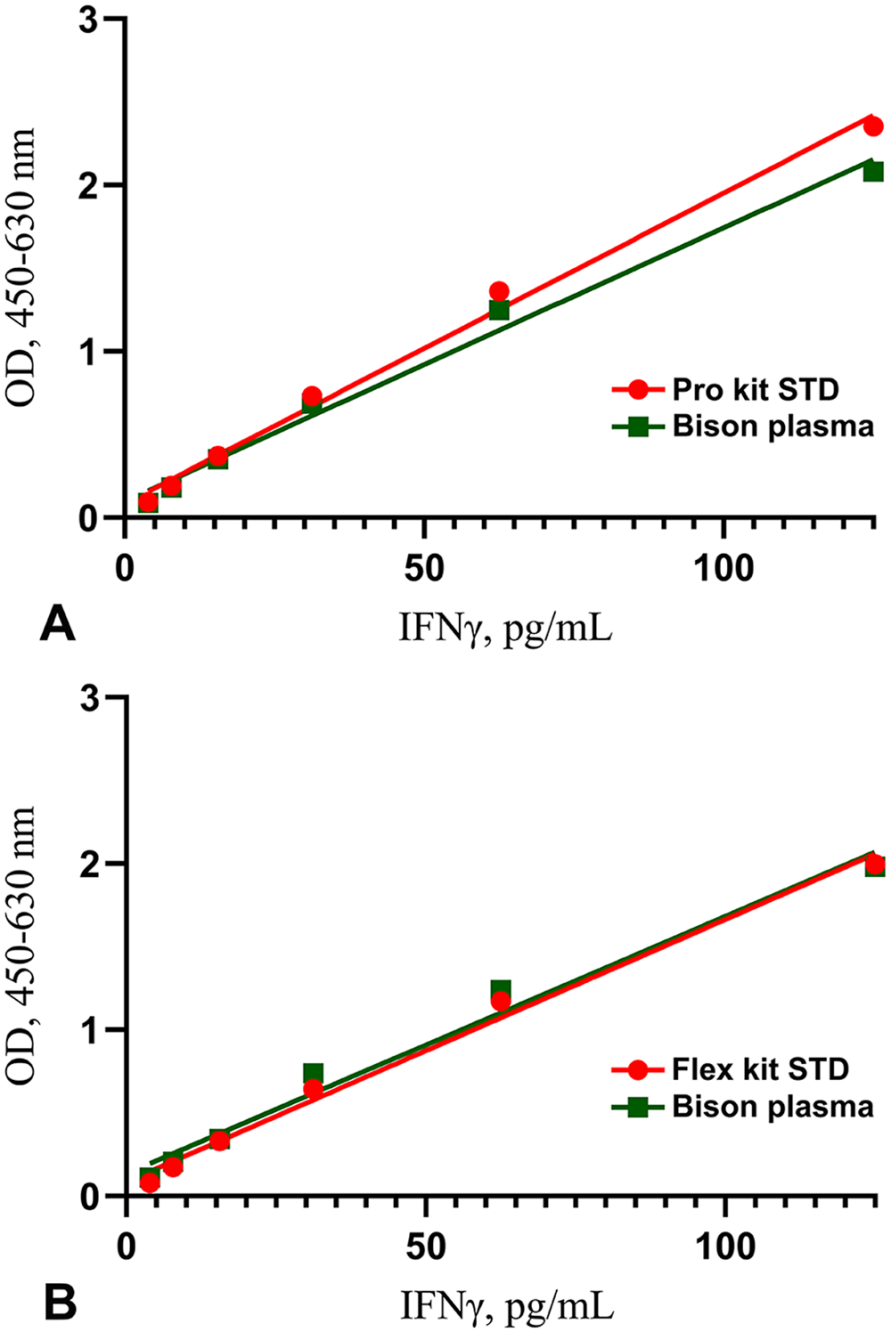
Regression analysis of dilution series of recombinant bovine interferon-gamma (IFNγ) and bison IFNγ in plasma of 1–125 pg/mL, measured with the **A)** Pro kit and **B)** Flex kit. In both ELISAs, samples had linear responses (*R*^2^ > 0.99) with no significant difference between lines (*F* = 3.673; *p* > 0.05). OD = optical density.

The Flex and Pro kit linear regression response was not significantly different from that of endogenous bison IFNγ (*R*^2^ > 0.99; *F* = 3.673, *p* > 0.05; Fig. 2). Recovery of rIFNγ in pooled nil bison plasma after serial dilution in ELISA diluent (1:2–1:32) for the Flex kit was 101–117% with a mean of 112%. Similarly, in the Pro kit, the recovery was 84–93% (x̄ = 91%).

Intra- and inter-assay reproducibility were determined using 4 different bison plasma samples; for the Flex kit, intra- and inter-assay CVs were 0.1–2.5% and 7.6–10%, respectively. For the Pro kit, intra- and inter-assay CVs were 1.3–3% and 0.3–8.7%, respectively (Table 1).

**Table 1.** Intra- and inter-assay reproducibility of pokeweed mitogen–stimulated whole blood from 4 bison diluted in pooled nil plasma in triplicate for 3 different days using the Flex kit and Pro kits.

	Intra-assay			Inter-assay		
	x̄, pg/mL	SD	CV%	x̄, pg/mL	SD	CV%
Pro kit						
Animal 1	115	2.4	2.1	117	2.7	2.3
Animal 2	119	1.5	1.3	121	3.5	2.9
Animal 3	87	1.1	1.3	93	8.1	8.7
Animal 4	81	2.4	3.0	81	0.2	0.3
Flex kit						
Animal 1	102	0.1	0.1	105	9.6	9.2
Animal 2	105	0.9	0.9	107	10.7	10.0
Animal 3	75	1.6	2.1	81	6.1	7.6
Animal 4	76	1.9	2.5	79	6.5	8.2

The Flex and Pro kit LOD and LOQ were both calculated as 1 pg/mL, respectively. Although this is the lowest concentration that can be detected, it does not guarantee precise and accurate quantification. Therefore, to ensure reliability and reproducibility in results, in subsequent runs we adopted the recommended LOQ by the manufacturer of 2 pg/mL for the Flex kit, and 3.16 pg/mL for the Pro kit.

**Figure 3.**
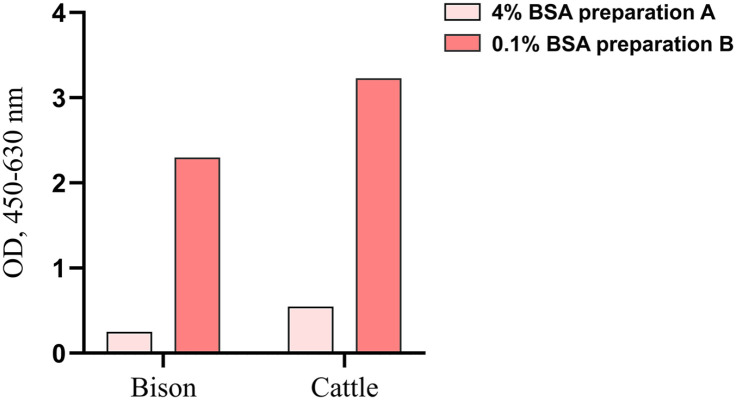
Bar graph of pooled pokeweed mitogen–stimulated plasma samples from bison and cattle, diluted 1:2 and assayed using the Bio-Rad kit and blocked with the following BSA preparations: 1× PBS with 4% BSA as recommended by the manufacturer and 1× PBS with 0.1% BSA (a modified protocol). Results from the modified protocol displayed the greatest optical density (OD).

We also investigated the effect of different BSA preparations for the detection of bison IFNγ using the Bio-Rad kit; the BSA preparation used in the modified protocol (1× PBS with 0.1% BSA) had a stronger IFNγ signal compared to the BSA preparation (1× PBS with 4% BSA; Fig. 3) recommended by the manufacturer.

## Discussion

We evaluated 3 antibody pairs and 2 precoated ELISAs for the detection of bison IFNγ. Both the Flex and Pro kits were more sensitive than the Bio-Rad and Bovigam kits. The Flex and Pro kits had a stronger signal than the Bio-Rad and Bovigam kits, with good recovery of endogenous IFNγ in the bison plasma, great linearity of dilution, and excellent reproducibility. Furthermore, using the BSA preparation of 1× PBS with 0.1% BSA resulted in improved bison and cattle IFNγ detection following a modified protocol of the Bio-Rad bovine IFNγ-specific ELISA kit. Therefore, we selected the Flex kit and Pro kit to measure bison IFNγ.

That the Flex and Pro kits were more sensitive than the Bio-Rad and Bovigam kits suggests that the ability of antibodies to cross-react between different species, in this case cattle and bison, can influence the accuracy of analyte detection [Committee for Medical Products for Human Use. ICH harmonization for better health. Guideline on validation of analytical procedures: text and methodology Q2(R2). 2023 Dec 14. Amsterdam, The Netherlands. https://www.ema.europa.eu/en/documents/scientific-guideline/ich-q2r2-guideline-validation-analytical-procedures-step-5-revision-1_en.pdf].

The selection of the bovine IFNγ antibody pair by Mabtech in an ELISA platform for measuring bison IFNγ was as predicted, given the ability of these antibodies to cross-react with other species, such as sheep and goat (https://www.mabtech.com/products/3119-6). Furthermore, our results demonstrate that these antibodies improve sensitivity by minimizing nonspecific binding, as clearly shown by the distinction between PWM and nil responses. Assay performance and accuracy can be interfered with by molecules in the sample, particularly in complex heterogeneous biological matrices such as plasma.^19^ We used the Mabtech ELISA diluent, which prevents heterophilic interferences in serum and plasma samples and improved the Bio-Rad kit performance in measuring bison IFNγ, which agrees with other studies that demonstrated using acid and heat improves assay sensitivity without compromising specificity.^14^ Furthermore, the use of IFNγ ELISAs in the detection of *M. bovis* in wildlife species has proven useful.^3,5,12^

Using blocking buffer with 1× PBS with 0.1% BSA had a stronger signal for bison IFNγ in plasma compared to 1× PBS with 4% BSA in the Bio-Rad kit. Although BSA is a well-established blocking agent known for reducing nonspecific protein-surface interactions in solid-phase immunoassays,^11^ the observed difference in signal suggests a complex interaction between blocking efficiency and analyte detection. At lower concentrations, such as 0.1% BSA, reduced blocking efficiency may increase nonspecific binding, potentially contributing to a stronger signal. However, it is equally plausible that lower BSA concentrations reduce steric hindrance, thereby enhancing the accessibility of the specific analyte or detection antibody to the target, ultimately amplifying the signal. We used negative assay controls to ensure that the test well signal was specific to the PWM plasma sample and normalized OD values. The inclusion of negative controls confirms that the observed stronger signal is empirical and not an artifact of nonspecific binding. Our findings underscore the importance of carefully optimizing BSA concentration in ELISA protocols to strike a balance between minimizing nonspecific interactions and maximizing specific binding for reliable assay performance.^24^

Lot-to-lot variability can be a disadvantage associated with using BSA as a blocking agent.^11,24^ Another reason why using 1× PBS with 4% BSA resulted in the detection of a much lower bison IFNγ signal might have been that the surface of the microtiter wells became oversaturated with BSA, especially because the recommended BSA concentration is 1–3%.^11^ This highlights the importance of ensuring that the best reagents and methods for an ELISA are optimized and is a reminder that not all BSA preparations will have reproducible results; hence, researchers must reference product lot numbers in their methods.^24^

The Flex and Pro kits performed the best in measuring bison IFNγ in plasma samples. Bison plasma showed minimal interference with the ELISA, and the linearity of dilution recovery was within the acceptable range of 80–120% [Guideline ICH Harmonised Tripartite. Validation of analytical procedures: text and methodology Q2(R1). Proc Int Conf Harmonization of Technical Requirements for Registration of Pharmaceuticals for Human Use; Nov 2005; Geneva, Switzerland. https://www.ich.org/fileadmin/Public_Web_Site/ICH_Products/Guidelines/Quality/Q2_R1/Step4/Q2_R1Guideline.pdf].

Furthermore, parallelism was excellent with a linear response and LOQ for both ELISAs, which highlights the utility of the assays for measuring bison IFNγ in plasma samples.^22^ Although the calculated LOQ of 1 pg/mL satisfies the mathematical threshold, achieving consistent accuracy and precision at this level may present practical challenges. Therefore, to ensure the reliability and reproducibility of results, we used the recommended LOQ because of the increased reliability of the results; employing a higher LOQ improves accuracy and precision. Moreover, the manufacturer’s extensive testing and rigorous validation processes further support the use of the recommended LOQ to guarantee reproducibility and reliability. It is important to note that, although the LOQ may be equivalent to the LOD, it cannot be lower and may often be set at a higher concentration to ensure robust performance.^2,6,17^

For both ELISAs, the intra- and inter-assay precision CVs of <10% and <15%, respectively, are within the range recommended by the Guideline ICH Harmonised Tripartite. Our results agree with a study that showed excellent intra- and inter-assay reproducibility of the Pro kit in African buffalo.^19^ Furthermore, studies have shown the successful use of the Mabtech IFNγ antibodies to measure IFNγ in different African wildlife species.^5,12,18^ Hence, both Flex and Pro kits are well-suited and reliable for quantifying bison IFNγ levels under specific operating procedures and offer flexibility depending on available laboratory resources. Our findings may advance bTB research, and further research is underway to investigate the performance of the Mabtech bovine IFNγ assay as a test for detecting *M. bovis* infection in infected bison populations. Although our findings provide valuable insights, the small sample size (*n* = 4) is a limitation that should be acknowledged; further research is needed with a large sample size.
